# COVID-19 risks in private equity nursing homes in Hesse, Germany – a retrospective cohort study

**DOI:** 10.1186/s12877-023-04361-8

**Published:** 2023-10-11

**Authors:** Janis Evers, Max Geraedts

**Affiliations:** Institute for Health Services Research and Clinical Epidemiology, Philipps-Universität Marburg, Marburg, Germany

**Keywords:** Private-equity-owned nursing homes, COVID-19 infections and deaths, Geriatric long-term care facilities, Staff qualifications, Proportion of single rooms

## Abstract

**Background:**

Private-equity-owned nursing homes (PENH) represent the strongest form of profit orientation in the nursing care market. Private equity firms aim to increase the profitability of nursing care facilities, which often leads to cost-cutting measures and the use of less qualified staff. Our study aims to fill the existing knowledge gap by examining the association between private equity ownership and COVID-19 related infections and deaths among residents and staff during the COVID-19 pandemic.

**Methods:**

We analyzed outbreak and mortality data for the period from 20/03/2020 to 05/01/2022 from 32 long-term care facilities in the Federal State of Hesse, Germany, which included 16 PENH that were propensity score matched on regional population density and number of beds with 16 non-PENH. We used logistic regression to determine the odds ratios (OR) for above-median values for the independent variables of PENH-status, number of beds, proportion of single rooms, registered nurses' ratio, and copayments.

**Results:**

PENH had substantially fewer outbreaks in number, but longer and larger outbreaks among nursing home residents, as well as a markedly increased proportion of deceased residents. The odds of the outcome "infections & deaths" were 5.38 (*p* <. 05) times higher among PENH compared to non-PENH.

**Conclusions:**

The study indicates a need for further research into the quality of care in PENH to inform evidence-based policy decisions, given the higher infection and death rates. Improved documentation and public visibility of PENH is also recommended, in line with existing practices for for-profit and non-profit nursing homes in Germany. Given our findings, regulatory bodies should closely observe PENH operational practices.

## Background

Nearly seven million people have died since the global outbreak of SARS-CoV-2 (COVID-19) in spring 2020 [1]. Nursing home residents were disproportionately affected [2, 3], with differences in the outcomes between for- and non-profit homes [4–6].

Private-equity-owned nursing homes (PENH) represent the strongest form of profit orientation in the nursing care market. Private equity refers to investments made in privately held companies, where capital is provided by private equity firms or investors. Unlike non- or other for-profit nursing homes, private equity firms acquire ownership stakes in these companies, with the intention of generating returns through various strategies such as restructuring, operational enhancements, and sale of the facility. Private equity investments are characterized by their focus on maximizing profitability and often involve implementing cost-cutting measures and optimizing operational efficiencies [7]. Private equity-companies have shown a heightened interest in the German nursing care market in recent years [8, 9], but their performance during the COVID-19-pandemic in Germany is widely unknown.

The increasing demand for nursing care is accompanied by an increasing volume of revenue for private equity companies [7, 10]. Private equity companies aim to increase the profitability of nursing homes, which often leads to cost-cutting measures and the use of less qualified staff [7, 11].

Staff is an associated risk factor due to regular external contacts [12, 13], which is moderated by the registered nurses’ ratio and number of staff members [5, 14, 15] and has been associated with fewer deaths by COVID-19 when there is a higher registered nurses’ ratio [14, 16]. In Germany, a registered nurses’ ratio of 50% for qualified personnel is mandated; however, not all facilities are able to meet this requirement due to a shortage of skilled staff. We used the registered nurse ratio provided by the Nursing Care Inspectorate of Hesse, based on annual reports from nursing homes. Another risk factor are shared rooms, as they make it difficult to isolate sick residents and lead to further transmissions of the pandemic pathogen [4, 16, 17]. In Germany, there are minimum construction requirements for medical facilities and nursing homes, such as those related to accessibility, the number of multi-bedrooms, and room sizes. However, it is not always ensured that these requirements are correctly implemented [18].

To date there is scant knowledge about the relationship between private equity ownership and the performance of nursing homes during the COVID-19 pandemic. According to an US-study no differences were observed between PENH and non-PENH regarding outbreaks and infections [19], while a Canadian study found higher death rates in PENH [20]. We hypothesize that PENH try to accommodate more residents in fewer rooms and have a lower registered nurses’ ratio which leads to poorer outcomes regarding the protection of residents and staff due to their expected profit maximization. Our aim was to assess the performance of PENH during the COVID-19 pandemic in Germany, specifically in terms of the number of infected and deceased residents, as well as infected staff, arising from private equity ownership. This was intended to provide policymakers with an opportunity to act.

## Methods

The study data cover the period from 20/03/2020 to 05/01/2022. The data was provided by the Nursing Care Inspectorate of Hesse and the Federal Association of the AOK, Germany’s largest health insurer. This data was collected by the authority for outbreak oversight purposes. Outbreaks in nursing homes are defined as situations where at least one staff member or resident has become infected. These outbreaks were initially reported to the Nursing Care Inspectorate of Hesse. The outbreak is considered over when there are no more infected staff members or residents. Once the outbreak ends, the authority is informed about the total number of infected individuals and deaths. All individuals who have died from or with a COVID-19 infection, regardless of the location of death, are considered deceased in this study.

In our retrospective cohort study, using this secondary data, we formed a subset of 16 uniquely identifiable PENH, which represented the only PENH that we were able to identify from the data. We identified the PENH facilities through preliminary work conducted by an expert [8, 9] and a multi-step internet search process. Initially, a keyword search combining the known private equity operators and the location of nursing homes in Hesse was performed. This search provided direct links to the operators’ websites or newspaper articles, which were manually verified. Additionally, Google Timeline was utilized to reconstruct any acquisitions or changes in ownership. The PENH had to been under private-equity ownership administration since at least December 1, 2020.

These 16 PENH were matched with 16 non-PENH counterparts, resulting in a total of 32 pairs. The 16 additional facilities were assigned using propensity score matching (PSM) with IBM SPSS 29 [21] based on facility size and regional population density with nearest neighbor matching (1:1). We chose these variables to maintain consistency in terms of the scope of facility management and the distinction between urban and rural areas, such as factors related to mobility and social structure. Furthermore, we anticipated differences in the distribution of other variables, which is why they were not included in the matching process. To maintain comparability, we calculated average scores across all outbreaks for dependent variables (DV) infections and COVID-19 related deaths.

The data was obtained from a pool of 687 long-term care facilities out of a total of 879 in the Federal State of Hesse, which is one of the 16 German states. Hesse has a population of 6.3 million, accounting for approximately 7.6% of Germany’s total population. The DV number of outbreaks, duration of outbreaks, proportion of infected residents, proportion of infected staff, and proportion of deceased residents, were dichotomized along the median of the DV to determine the odds ratios (OR) for an above-median value for the independent variables (IV) of private equity-status, proportion of single rooms, registered nurses’ ratio, and copayments using logistic regression. Due to their characteristic lack of reliance on the assumption of normally distributed residuals, logistic regression models were utilized. To avoid overinterpretation, we dichotomized the data at the median, to prevent splitting at the optimal cutoff point.

Residents of nursing homes in Germany have to pay a copayment for their accommodation. The amount of the copayments depends on the resident’s level of care (LoC) and the facility prices, which depend on the equipment and services and are negotiated with long-term care insurances and the municipalities [22]. In addition to the LoC, the cost structure of nursing home facilities can be influenced by multiple variables such as personnel qualifications, the scope and quality of medical services provided, the profit-orientation, additional care services as well as regional discrepancies in rent and utility expenditures. If residents or their relatives are unable to meet the costs, social welfare funds can pay some or all of the costs for residents. In our study, we utilized the nursing care costs for Level of Care (LoC) 1, assuming that the basic costs are similar across all levels and that the increase from LoC 1 to higher levels follows a linear progression. The significance level of the study was set at *p* < .05 and we found no evidence of multicollinearity between the IV. All calculations were performed with IBM SPSS 29.

## Results

Of the 16 non-PENH, 10 were run by for-profit providers. The average number of beds was 113 for PENH and 115 for non-PENH. The average regional population density was 68,644 per county for PENH and 83,984 for non-PENH. The average percentage of registered nurses in the sample was 46.8%, which was 3.2% below the legally required minimum standard in Germany of 50%. The proportion of single rooms in these facilities, at 52.3%, was also lower than the Hessian state average of 65.4%. Table 1 provides a detailed breakdown of the data. We conducted a balance check using a t-test and can confirm that the two groups do not significantly differ in terms of the PSM variables.

**Table 1:** Descriptive statistics of the independent and dependent variables for all 32 nursing homes under study

	Average	Minimum	Maximum	Standard deviation
Regional population density	76,314	2977	764,104	187,347
No. of beds	114	26	173	44
Single rooms (%)	52.3	1.7	100	30.1
Registered nurses’ ratio (%)	46.8	33.1	56.2	6.0
Copayment € (LoC1)	2435.7	1928.4	3563.7	324.4
Duration of outbreak (days)	36.8	0	105	23.7
No. of outbreaks	2.7	0	8	2.26
Infected residents (%)	20.8	0	103.6	29.2
Infected staff (%)	9.2	0	82.8	15.6
Deceased residents (%)	5.4	0	35.3	9.2

*No*. Number, *LoC *Level of care

Table 2 depicts the structural differences and the infected residents and staff, as well as the deceased residents for PENH and non-PENH. The PENH and non-PENH revealed an almost similar facility size, proportion of single rooms, and registered nurses’ ratio as well as detailed information for the median split of the DVs. The PENH had substantially fewer outbreaks in number, but with longer and larger outbreaks among residents, as well as a markedly increased proportion of deceased residents.

**Table 2:** Average structural characteristics of nursing homes and infected residents and staff and deceased residents by PE-Status with median split of the dependent variables and number of nursing homes over median (PENH: *N* = 16. Non-PENH: *N* = 16)

Category	PENH (AVG, *N* = 16)	Non-PENH (AVG, *N* = 16)	Median (depended Variables only)	PENH over Median
Number of beds	113	115		
Single rooms (%)	52.1	52.5		
Registered nurses’ ratio (%)	47	46.7		
Copayment € (LoC 1)	2359.5	2511.8		
For-Profit (%)	100	56.3		
Outbreaks (No.)	2	3.38	2	7
Duration (days)	47.1	26.4	34.8	11
Infected residents (%)	27.8	13.8	5.5	11
Infected staff (%)	9.3	9.1	3.8	11
Deceased residents (%)	8.5	2.3	1.3	11

*PENH* Private-equity-owned nursing homes, *LoC *Level of care, *No*. Numbers

The 16 facilities with above-median values for infected residents and staff, as well as deceased residents, are the same facilities. Therefore, the result is reported only once as infections & deaths and applies to both infection-related scores as well as deceased residents depicts.

Table 3 presents Odds ratios (OR) resulting from a logistic regression analysis of the DVs: number of outbreaks, duration of outbreaks, and infections & deaths. The analysis adjusts for several facility characteristics, PE-status, registered nurses’ ratio, proportion of single rooms, and copayments. The odds for the outcome “infections & deaths” were 5.38 times higher among PENH compared to non-PENH when controlling for the other facility characteristics (*p* < .05). There was no other significant result. According to the recommendation of Backhaus et al. [23] the models of duration of outbreaks (*R*²=0.201) and infections & deaths (*R*²=0.244) had an acceptable variance explanation, while the variance explanation for the model of number of outbreaks was good (*R*²=0.418).

**Table 3:** OR from logistic regression analysis for the median-dichotomized variables number of outbreaks, duration of outbreaks, and infections & deaths, adjusting for facility characteristics

Independent Variable	OR	LCI	UCI	p
Number of outbreaks				
PENH	0.25	0.04	1.56	0.14
Registered nurses ‘ratio	1.23	0.96	1.31	0.14
Proportion of single rooms	1.0	0.97	1.4	0.80
Copayments	1.01	1.0	1.01	0.08
Duration of outbreaks				
PENH	4.61	0.98	21.62	0.053
Registered nurses ‘ratio	1.01	0.89	1.15	0.88
Proportion of single rooms	1.01	0.98	1.04	0.47
Copayments	0.99	0.99	1.0	0.68
Infections & deaths				
PENH	5.39	1.1	26.6	0.04
Registered nurses ‘ratio	1.06	0.92	1.22	0.41
Proportion of single rooms	0.99	0.94	1.03	0.76
Copayments	1	0.99	1.0	0.88

*PENH* Private-equity-owned nursing homes, *OR *Odds ratio, *LCI *Lower confidence interval, *UCI *Upper confidence interval, *p p*-value

## Discussion

Our study found that the proportion of nursing home residents infected with or deceased from COVID-19 during 20/03/2020 to 05/01/2022 was significantly higher in PENH than in non-PENH in Hesse, Germany. Our initial assumption of differences in the registered nurses’ ratio and the proportion of single rooms could not be confirmed, leading us to conclude that other factors that were not measured must be responsible for the large differences between PENH and non-PENH regarding infections & deaths. The relatively low infection rates among staff compared to residents may suggest that PENH were less diligent in testing their employees, leading to longer and larger outbreaks among the residents. Differences in the actual qualifications of registered nurses or a work environment that promotes the spread of the pandemic are also possible [11]. For example, it is conceivable that registered nurses may undertake organizational tasks in addition to their own care work, resulting in less care time per resident. Care time per minute is an important factor to regulate COVID-19 outbreaks [15], unfortunately, we have no data on this matter. The results of our study regarding the number of infected and deceased nursing home residents are different from those reported in an US-study [19]. Possible discrepancies in the findings may be attributed to variations in the study populations and the duration of the observation period. In our study, although the observation period was longer, we had a smaller sample size. To examine the effects of PENH on patient care during a pandemic, qualitative and, as suggested by the US-Study longitudinal studies should be conducted.

A limitation of our study is the small sample size, which may have compromised the statistical power of our results. The small sample size was due to the fact that the identification of PENH was challenging since there is no registry available in Germany. We conducted an internet search using the provider pages of PE-chains operating in Germany and monitored purchases and sales. Furthermore, all results were manually verified and validated by an expert. However, it is possible that some PENH in Hesse may not have been identified. This study is subject to the limitations inherent to secondary data analysis, including potential issues with data quality, uncertainties about the methodologies used during data collection, and our lack of control over the initial data gathering process.

Furthermore, it is possible that differences in the spread of the virus within the facilities may have occurred due to structural variations of the building resulting from the construction year of the facilities. Unfortunately, we were unable to examine this aspect with the available data. Therefore, the generalizability of the findings to the overall population may be limited. Since we split the outcome variables into two groups using the median as the dividing point, the results can only suggest trends for the group with values higher than the median. Thus, the results do not indicate the total increased risk, but the probability of a greater than median expression. The mortality rate associated with COVID-19 infection is largely influenced by the age and comorbidity burden of the residents [24]. Due to delay in data collection during the pandemic and privacy concerns, we do not have access to this information. In Germany, the mortality rates in relation to COVID-19 have been found to vary based on social determinants [25, 26]. This may be attributed to various factors, including the selection of nursing homes, which could be influenced by the availability of facilities and the financial resources of residents and their families. Additionally, these factors may be interconnected with social determinants or the demographic and social structures of specific districts or counties. Thus, we performed propensity score matching based on population density to account for potential differences between urban and rural populations. We assume that age and disease burden are distributed similarly in both groups. We assess the validity of the DV as sufficient for our survey since our aim was to identify differences between PENH and non-PENH and not to replicate exact infection courses. Sensitivity analyses were conducted for the logistic models, confirming a good sensitivity of 68.8–81.3%. Despite the limitations mentioned, our study indicates that PENH should be extensively scrutinized.

## Conclusion

Our findings reveal that PENH have significantly higher rates of COVID-19 infections and deaths among residents compared to non-PENH. This raises serious concerns about the quality of care in these privately-run, for-profit nursing homes, which are the most profit-driven facilities in the care market. By exploring the underlying factors that contribute to the higher infection and death rates in PENH, officials can develop evidence-based policies that prioritize the well-being and safety of nursing home residents and staff. One possible explanation for these elevated rates could be less diligent testing among staff in PENH, which may lead to larger and more prolonged outbreaks among residents. Another area of concern is the qualifications and work environment of registered nurses in PENH, which may not be up to the standard required to control infectious diseases like COVID-19. Our findings suggest that regulatory bodies should closely observe the operational practices within PENH. Due to the differences found, authorities should improve their documentation and make PENH publicly visible, as is already the case for for-profit and non-profit nursing homes in Germany. Given the small sample size of our study, further research in this area is necessary. Understanding the relationship between PENH and quality of care is essential to provide policymakers with better options for action.

## Data Availability

The data that support the findings of this study are available from Hessische Betreuungs- und Pflegeaufsicht but restrictions apply to the availability of these data, which were used under license for the current study, and so are not publicly available. Data are however available from the authors upon reasonable request and with permission of Hessische Betreuungs- und Pflegeaufsicht.
